# Increased water salinity applied to tomato plants accelerates the development of the leaf miner *Tuta absoluta* through bottom-up effects

**DOI:** 10.1038/srep32403

**Published:** 2016-09-13

**Authors:** Peng Han, Zhi-jian Wang, Anne-Violette Lavoir, Thomas Michel, Aurélie Seassau, Wen-yan Zheng, Chang-ying Niu, Nicolas Desneux

**Affiliations:** 1 College of Plant Science & Technology, Huazhong Agricultural University, Wuhan 430070, China; 2 INRA (French National Institute for Agricultural Research) Univ. Nice Sophia Antipolis, CNRS, UMR 1355-7254 Institut Sophia Agrobiotech, 06903 Sophia-Antipolis, France; 3 Institut de Chimie de Nice, Université Nice Sophia-Antipolis, UMR CNRS 7272, Parc Valrose, F-06108 Nice, France

## Abstract

Variation in resource inputs to plants may trigger bottom-up effects on herbivorous insects. We examined the effects of water input: optimal water *vs*. limited water; water salinity: with *vs.* without addition of 100 mM NaCl; and their interactions on tomato plants (*Solanum lycopersicum*), and consequently, the bottom-up effects on the tomato leaf miner, *Tuta absoluta* (Meytick) (Lepidoptera: Gelechiidae). Plant growth was significantly impeded by limited water input and NaCl addition. In terms of leaf chemical defense, the production of tomatidine significantly increased with limited water and NaCl addition, and a similar but non-significant trend was observed for the other glycoalkaloids. *Tuta absoluta* survival did not vary with the water and salinity treatments, but the treatment “optimal water-high salinity” increased the development rate without lowering pupal mass. Our results suggest that caution should be used in the IPM program against *T. absoluta* when irrigating tomato crops with saline water.

Plants are known to serve as a food source and shelter, thus ensuring survival and development of herbivorous insects^1^, although plants adopt various defensive strategies to cope with insect herbivory^2^,^3^. Plants, however, often grow in a variable environment where abiotic stress could be caused by cold, heat, drought, salt or chemical pollutants^4^. The environmental abiotic factors trigger changes in plant characteristics which can subsequently impact the performance of herbivorous insects. Such a cascading effect through plant-insect interactions is called the bottom-up effect^5^,^6^,^7^.

Water is crucial for plant growth, and water limitation can cause considerable changes in plant morphology, physiology and biochemistry^8^. Plants under drought conditions often impede survival and development of herbivorous insects because of the enhanced plant chemical defense^9^, and/or decreased nutritive quality of host as food^6^,^10^. However, there is no general consensus on the effects of water limitation on herbivorous insects since positive, negative and non-significant effects have all been documented^11^. Various factors have been shown to mediate the diversity of responses, such as the pattern of water limitation^11^, the feeding strategy adopted by insects^6^, e.g., chewing or sap-feeding insects, as well as feeding specialization^9^, e.g., specialist or generalist.

Plant-water relationships are often mediated by salinity status of irrigation, particularly in agro-ecosystems^12^. Salinity stress is considered a major environmental issue and a substantial constraint to plant growth^13^. One of the mechanisms may be that plants face lower water availability because of the increased salt concentration in the irrigation water^12^,^14^. Sodium chloride (NaCl) is one of the most common ingredients in soil or irrigation water causing salinity stress in plants^4^. NaCl stress can induce loss of intracellular water in plants. In addition, the effects of salinity on nitrogen metabolism are highly relevant since it may reflect osmotic and/or specific interactions of NaCl in several steps of nitrogen assimilation^13^. Increased soil salinity may induce changes in plant quality, especially in secondary metabolism^15^; these changes may in turn have an impact on herbivorous insects through bottom-up effects. However, little information is known about such bottom-up effects except those studies conducted in salt marsh systems where insects have been found to be positively^16^,^17^,^18^, negatively^19^, or neutrally^20^,^21^ influenced by increasing soil salinity. These differing results could be explained by the impact of variation in salinity stress tolerance in plants^18^, variation in predation pressure due to plant morphological characteristics^17^, as well as other abiotic features such as nitrogen fertilization^21^.

Unlike natural ecosystems where many salinity-tolerant species have evolved to adapt to high salinity^22^, many acclimated crop cultivars are not salinity-tolerant. This is especially true for those grown under controlled cropping systems, e.g., greenhouses. In these systems, economically dependent on certain leaf-mining insects, herbivory by pest insects has long been a major agricultural challenge^10^. The larvae can penetrate and feed within the plant tissues and they are thus considered to have intimate relationship with their host plants^6^. However, the bottom-up effect of salinity stress on leaf-mining insects has rarely been documented. Moreover, the potential interactive effect of water and salinity stress on leaf-mining insects remains elusive.

With this context in mind, we examined the bottom-up effects of water and salinity on a leaf-mining insect in an agro-ecosystem. We evaluated the effects of water-salinity on plant growth and chemical defense traits, as well as on insect survival and development. Since strong bottom-up effects of soil nitrogen and water inputs on the performance of a leaf miner have been reported owing to the changes in plant nutritional quality as well as plant chemical defense^10^, we hypothesized that varying soil water/salinity may also trigger bottom-up effects on the leaf miner. To test this hypothesis, we set up a “water/salinity – plant – leaf miner” system using the tomato plant, *Solanum lycopersicum* L and the tomato leaf miner, *Tuta absoluta* Meyrick (Lepidoptera: Gelechiidae). Tomatoes are important greenhouse crops in many semi-arid regions where soil and groundwater salinity have been considered a major issue in crop production such as the Mediterranean region^12^,^23^. *Tuta absoluta* is a devastating pest threatening the worldwide tomato production^24^,^25^,^26^. This species has invaded Europe, rapidly spreading across the Mediterranean basin countries since its first appearance in Spain in 2006^27^. Since the invasion, the “tomato – leaf miner” system has been extensively studied^28^,^29^,^30^,^31^, notably in plant-insect interaction studies^10^,^32^.

## Results

### Plant growth

#### Plant height

**Figure 1:**
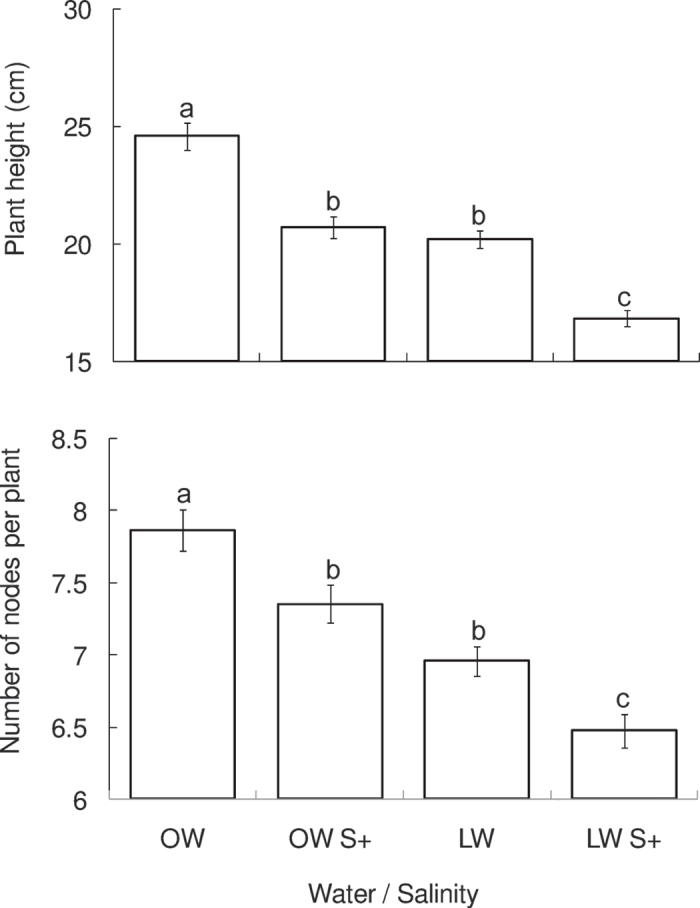
Plant height (mean ± SE, n = 19) and number of nodes (mean ± SE, n = 19) per plant on 35 DAS (days after sowing) treated with different water and salt inputs. OW: optimal water; LW: limited water; OW S+: optimal water and salinity stress (100 mM NaCl); LW S+: limited water and salinity stress. Histograms with different letters indicate significant difference at *P* < 0.05.

Tomato plant height measured on 34 DAS (days after sowing) was significantly affected by water and salt treatments (Table 1A). No interaction of the two factors was found. In comparison to OW (optimal water) treatment, plant height decreased by 15.8%, 17.9% and 31.6% under the OW S+ (optimal water with high salinity), LW (limited water) and LW S+ (limited water with high salinity) treatments, respectively (Fig. 1).

#### Number of nodes

The number of nodes per plant showed a similar response pattern to water and salt treatments (Table 1A and Fig. 1). This trait differed significantly among the four treatments averaging 7.9, 7.4, 7.0 and 6.5 under OW, OW S+, LW and LW S+, respectively (Fig. 1).

### Plant defense

**Figure 2:**
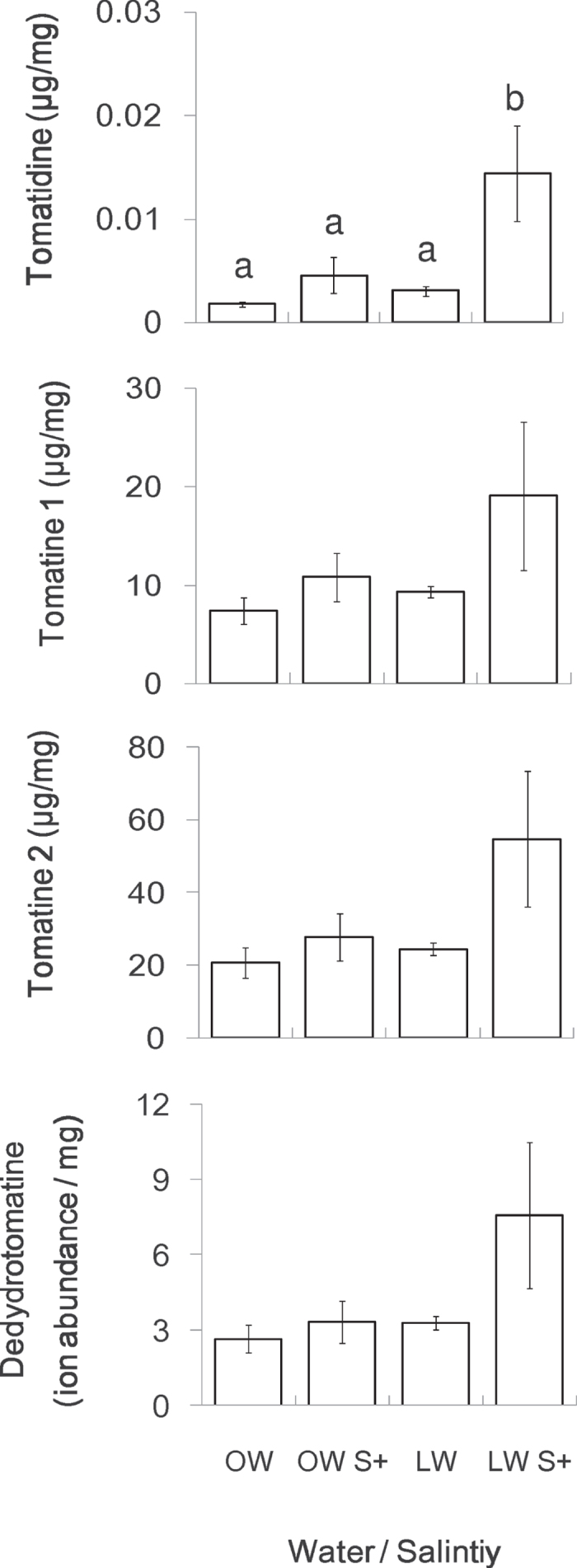
Effects of water and salt inputs on the concentrations of four glycoalkaloids in tomato leaves: tomatidine (μg/mg leaf dry mass (LDM)), α-tomatine 1 (μg/mg LDM), α-tomatine 2 (μg/mg LDM) and dehydrotomatine (x10^4^) (relative content: ion abundance/mg LDM). OW: optimal water; LW: limited water; OW S+: optimal water and salinity stress (100 mM NaCl); LW S+: limited water and salinity stress. Histograms with different letters indicate significant difference at *P* < 0.05. Absence of letters indicates no significant difference among water and salinity treatments.

Factorial ANOVAs suggested no significant effect of water, salt and their interactions on glycoalkaloids, except for one compound: tomatidine (Table 1A). The concentration of tomatidine increased significantly under LW S+ treatment compared to OW treatment, with intermediate levels for the other two treatments OW S+ and LW (Fig. 2). For the concentrations of the other three compounds measured, no marked difference was found among salinity-water treatments (despite similar trends than the one observed for tomatidine). The lowest level was obtained from the OW treatment, intermediate levels from the LW and OW S+, and the highest level from LW S+. *Tuta absoluta* herbivory did not induce changes in concentrations of the glycoalkaloids in leaves (“insect”: all *P* > 0.05 in Table 1A); therefore the data from the “With Tuta” and “Without Tuta” groups were pooled (Fig. 2).

### *T. absoluta* survival

**Figure 3:**
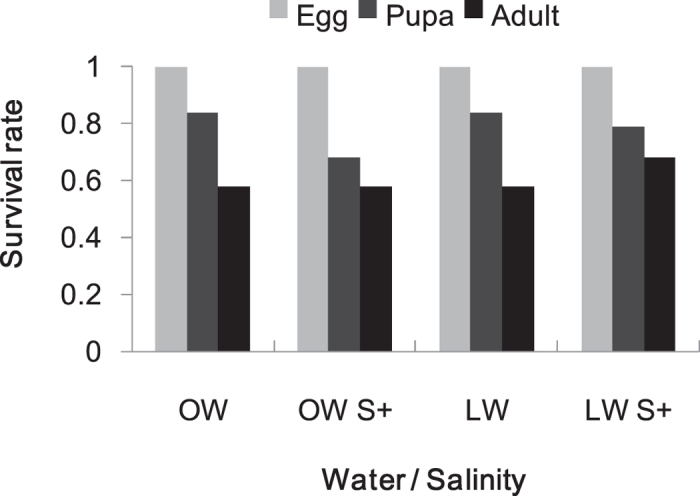
Survival rate of *T. absoluta* individual eggs reaching pupal and adult stage feeding on tomato plants treated with different water and salt inputs (n = 19). OW: optimal water; LW: limited water; OW S+: optimal water and salinity stress (100 mM NaCl); LW S+: limited water and salinity stress. Absence of letters indicates no significant difference in *T. absoluta* survival rate among water and salinity treatments (all *P* > 0.05, permuted Fisher exact test).

Neither water input nor salinity stress significantly affected *T. absoluta* survival rate (water: χ^2^ = 0.499, df = 1, *P* = 0.480; salinity: χ^2^  = 0.125, df = 1, *P* = 0.724; interaction: χ^2^  = 0.499, df = 1, P = 0.480) (Fig. 3). *Tuta absoluta* survival in response to water and salinity significantly depended on the developmental stages (stage: χ^2^  = 6.210, df = 1, *P* = 0.013). Overall, *T. absoluta* survival rate from egg to pupa or to adult did not differ among the four treatment combinations within each group (Fig. 3).

### *T. absoluta* development

#### Pupal weight

**Figure 4:**
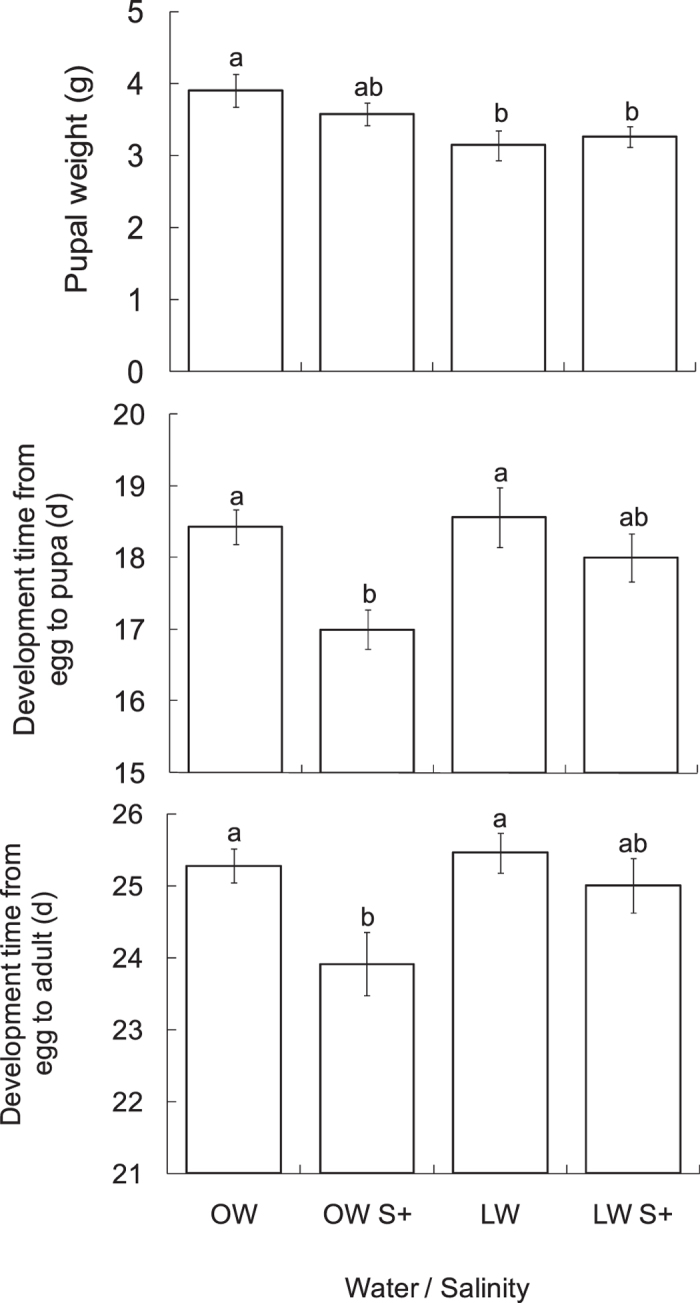
Pupal weight (mean ± SE, n = 13–15), development time from egg to pupa (mean ± SE, n = 13–16) and development time from egg to adult (mean ± SE, n = 11–13) of *T. absoluta* feeding on tomato plants treated with different water and salt inputs. OW: optimal water; LW: limited water; OW S+: optimal water and salinity stress (100 mM NaCl); LW S+: limited water and salinity stress. Histograms with different letters indicate significant difference at *P* < 0.05.

Water input significantly impacted *T. absoluta* pupal weight (Table 1B), whereas salt addition and its interaction with water input did not impact pupal weight. *Tuta absoluta* showed lower pupal weight on the plants treated with LW compared to the OW treatment (Fig. 4). The average pupal weight of the individual feeding on plants treated with OW, OW S+, LW and LW S+ was 3.91 g, 3.58 g, 3.15 g and 3.27 g, respectively.

#### Development time from egg to pupa or to adult

Salt addition had a significant effect on *T. absoluta* development time from egg to pupa or to adult (Table 1B), whereas water input and its interaction with salinity stress did not. While the development time from egg to pupa or to adult averaged 18.4 and 25.3 days under the OW treatment, *T. absoluta* exhibited shorter development times, i.e., 17.0 and 23.9 days under the OW S+ treatment, respectively (Fig. 4).

## Discussion

Our study demonstrated that varying water-salinity treatments on tomato plants triggered bottom-up effects on plant-leaf miner interactions. *Tuta absoluta* survival did not vary with the water and salinity treatments; however, the insect development rate increased without lowering pupal mass under the increased salinity conditions. Firstly, we demonstrated how plant growth and chemical defensive profiles were affected by various water and salinity treatments; then we explained how the changes in host plant nutritional and defensive features could explain the bottom-up effects on *T. absoluta*.

The four water and salinity treatments resulted in a gradient of plant growth performance with the OW-treated plants being the highest with the most nodes and LW S+ treated ones being the smallest with the fewest nodes (Fig. 1). Such a gradient has also been observed on tomato plants where their growth showed a gradient across six treatments in terms of physical damage, nitrogen and water inputs^6^. Reduced water availability to tomato plants restricted their growth, which corroborated our previous studies^10^,^33^. Besides the water itself, the effects of LW treatment on plants can also be attributed to a lower amount of nutrients in the solution. Salinity stress has also been shown to impede tomato plant growth (Fig. 1). High salinity usually inhibits photosynthesis by reducing plant water potential^22^. Similarly, many other studies have reported the direct negative impact of salinity stress on plant metabolism^4^,^12^,^22^,^34^, and an indirect one via disruptive use of other resources, e.g., nitrogen and water^13^,^35^,^36^,^37^,^38^.

While water shortage has been shown to induce higher accumulation of leaf glycoalkaloids in tomato plants^9^, to our knowledge, this is the first time that salinity stress has been found to have an impact on the production of these compounds in tomato leaves. A similar effect of salinity has been observed on glycoalkaloids in the roots of another plant species, *Catharanthus roseus*^39^. Moreover, high salinity has recently been documented to induce increased concentrations of other secondary metabolites in many plant species. The known examples are a) carotenoids, phenolics and antioxidative enzymes in lettuce^40^, b) phenolics, anthocyanins and flavones in sugarcane^41^, cyanogenic compounds in white clover^15^, c) various enzymatic defensive compounds in *Andrographis paniculata*^42^, d) diterpenes lactone, flavonoids and tannins in cotton^43^ and lastly, 1,4-benzoxazin-3-one aglycones in maize^44^. Increased production of these compounds could act to enhance chemical defense via their toxic activities^15^,^40^,^41^,^42^,^43^,^44^. In our study, however, the effect of water and salinity treatments was only seen on tomatidine even if a trend was observed for the other glycoalkaloids (Fig. 2). We assume the salt stress might be too weak to induce changes in glycoalkaloid concentrations in tomato leaves.

Although water shortage plus nutrient deficiency to plants (LW treatment) significantly reduced *T. absoluta* pupal weight, this treatment did not disrupt its development time. By contrast, *T. absoluta* development time was shortened when salt was added to the nutrient solution applied to plants (Fig. 4). We suggest that high soil salinity may influence *T. absoluta* development through three mechanisms. Firstly, salinity stress may affect *T. absoluta* development by reducing the availability of leaf water, since it can result in plant water deficit similar to a form of physiological drought^45^. In that case, *T. absoluta* larvae may face difficulties gaining sufficient water from tomato leaves. Secondly, salinity stress may affect the development of *T. absoluta* by enhancing the chemical defense of the host plants. Nevertheless, this hypothesis may not be supported by our data as the tomato plants did not produce more leaf glycoalkaloids under OW S+ treatment (Fig. 2) where a shorter development time in *T. absoluta* larvae was recorded (Fig. 4). Indeed, it has been suggested that glycoalkaloids are less concentrated and varied in cultivated plant types than in wild ones, as shown in the *Solanum* genus^46^. In our study, the only compound that varied with water and salinity, i.e., tomatidine, has been considered non-toxic for many herbivorous insects^47^. In addition, we explicitly acknowledge that tomatoes possess many other resistance-related traits that can impact their performance, including defensive compounds such as phenolics, e.g., chlorogenic acid, rutin and kaempferol and defense enzymes, e.g., polyphenol oxidase and protease inhibitor^28^,^48^. Thirdly, a possible explanation could be that excessive accumulation of Na^+^ and Cl^–^ ions^49^ and cyanide^15^ in leaves may lower the suitability of leaf as food for *T. absoluta* larvae. This hypothesis matches the results in Fig. 4 showing strong effects of salinity stress on *T. absoluta* development time under OW S+ treatment, whereas only a slight decrease was found under LW S+ treatment. Even with the same salt concentrations in both treatments, the absolute quantity of salts in OW S+ treatment was twice as high as in LW S+.

An interesting question to consider is why *T. absoluta* managed to reach a normal pupal mass despite the fact that they underwent shorter larval development period under NaCl stress (Fig. 4). It has been acknowledged that insects may pupate with a lower mass accumulation if they have shortened plant resource consumption under adverse conditions. One hypothesis could be that *T. absoluta* larvae have to compensate for low leaf water content by accelerating their feeding rate^1^. It appears that *T. absoluta* larvae were able to employ different feeding strategies to adapt to varying lower plant food suitability, which means taking more time for larval development to compensate for nitrogen deficiency^9^, while spending less time for larval development to cope with salinity stress in the present study. Another assumption is that the larvae may benefit from higher concentrations of amino acids present in leaves to speed up their development. Indeed, higher proteolytic activities have been recorded in tomato leaves when tomato plants received 100 mM NaCl for 10 days^13^.

In conclusion, salinity stress has triggered strong bottom-up effects on *T. absoluta* development. These results highlight the importance of considering the salinity in irrigation for tomato crops when an IPM package has been designed to manage *T. absoluta*. In practice, a shorter development time, but unaffected survival rate and pupal mass accumulation in *T. absoluta*, may raise concern regarding higher damage by this pest when the tomato plants are grown under saline conditions. We predict that *T. absoluta* may have a greater population increase potential owing to their shorter life-cycle. This is more likely to occur in regions where the ground water, relatively high in salinity, is often used to irrigate tomato plants^12^,^23^. Therefore, to provide a full understanding of salt – tomato – *T. absoluta* interactions, future work is needed to monitor population dynamics of *T. absoluta* in multiple generations. Furthermore, the arthropod biological control agents from the higher trophic levels, the parasitoids such as *Necremnus* sp., Trichogrammes and Braconidae species, and the predator such as *Macrolophus pygmaeus*, should be included in the testing system^26^. This is due to the fact that salinity stress in plants may trigger bottom-up effects on the tritrophic interactions “plant-herbivorous insect-natural enemy”. This has been shown on other types of resources such as nitrogen^29^,^50^,^51^.

## Methods

### Study organisms

**Figure 5:**
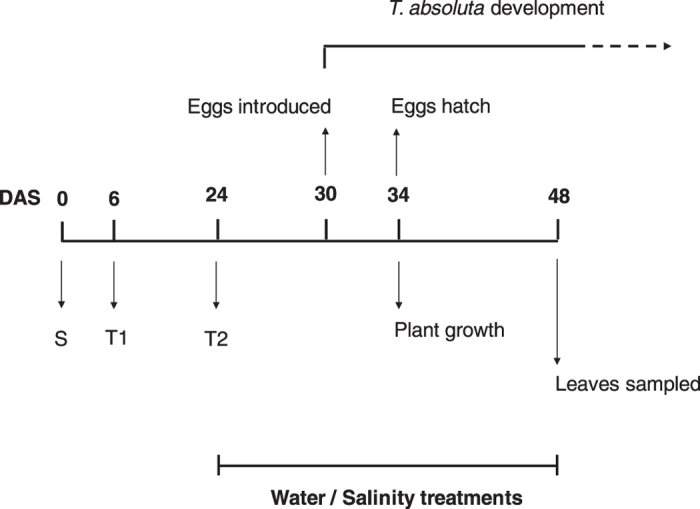
Experimental schedule: S: plant sowing; T1/T2: the first and second plant transfer; *T. absoluta* eggs introduction and development; plant growth measurement before herbivory by *T. absoluta* larvae; leaves sampled for glycoalkaloids quantification after *T. absoluta* larvae herbivory; DAS: days after sowing; Water and salinity treatments were performed from 24 DAS to 48 DAS.

The ‘Marmande’ tomato plant cultivar was grown in cubic plastic pots (7 × 7 × 6.5 cm) and kept in a climatic chamber (12 h light, 24 ± 1 °C, 65 ± 5% RH). Tomato seedlings were then transferred into new pots containing limestone grains (Perlite Italiana srl, Corsico, Italy) mixed with nutrient soil on 6 DAS (days after sowing) (Fig. 5). On 24 DAS, we transferred the plants into larger pots (diameter: 10 cm, height: 9 cm) filled only with limestone grains.

The *T. absoluta* colony was reared on tomato plants in cages (40 × 40 × 40 cm) and kept in a climatic chamber (16 h light, 25 ± 1 °C, 70 ± 10% RH). Honey and water were provided *ad libitum* as food source for *T. absoluta* adults. We collected 100 *T. absoluta* adults and put them into transparent plastic tubes (diameter: 3 cm, length: 10 cm) in order to gather the eggs for the subsequent experiments. Ten *T. absoluta* adults were released into each tube with one tomato leaflet put inside as the oviposition substrate. A total of ten tubes was prepared. Newly-oviposited *T. absoluta* eggs (≤24 h) were used to infest the plants.

### Water and NaCl treatments

We set up the water and NaCl treatments by manipulating the quality and quantity of the stock nutrient solution which had been regularly used by our team to rear tomato plants in climatic chambers. The formula of this stock solution was prepared by mixing and diluting the following three concentrated solutions in a 100 L reserve stock, respectively [Stock 1: HNO_3_ (58 g/L) in 3 L, H_3_PO_4_ (75 g/L) in 1.5 L; Stock 2: KNO_3_ = 7.5 kg, KH_2_PO_4_ = 3.5 kg, NO_3_NH_4_ = 0.5 kg, MgSO_4_ = 1.5 kg, HNO_3_ (58 g/L) in 50 mL, K_2_SO_4_ = 1 kg; Stock 3: KNO_3_ = 3.75 kg, Ca(NO_3_)_2_ = 12.5 kg, Masquolate Fe in 2.8 L, HNO_3_ (58% g/L) in 50 ml]. We carried out a full factorial design by combining the two levels of water treatment, i.e., optimal water *vs.* limited water input: “OW” *vs.* “LW”, and two levels of salinity treatment, i.e., with vs. without addition of 100 mM NaCl: “S+” *vs*. blank, to the plants starting on 24 DAS (Fig. 5). The final concentration of 100 mM in the nutrient solution was prepared to obtain S+ treatment as similar concentrations of NaCl had been used previously to create salinity stress on tomato plants^12^,^13^,^49^. For the S+ treatment, the base nutrient solution with 100 mM NaCl was applied to the plants on a daily basis starting from 24 DAS until the *T. absoluta* pupated (Fig. 5). In order to acclimatize the plants to the NaCl stress, the nutrient solution in 50 mM NaCl was applied on two consecutive days before the nutrient solution in 100 mM NaCl was initiated. The nutrient solution, without NaCl, was thus used as the control (“Blank”). To differentiate water input, the volume of two types of nutrient solution was supplied in a “step increase” pattern throughout the tomato growing stage, following the protocols from our previous studies^10^,^29^,^51^. Optimal daily water input, hereafter named “v” in volume of nutrient, was determined by the amount that fully saturates the perlite substrate without visible drainage, i.e., field capacity^10^. The limited water treatment was determined by irrigating the plants with v/2 of the nutrient solution^10^. We were unable to manipulate the water treatment by itself, thus it was accompanied by the simultaneous manipulation of nutrients inside the solution. In the latter case, the absolute quantity of nutrients in LW was half that in the OW treatment, but the concentration of nutrient solution remained constant among treatments. The LW treatment referred to half the volume of water as well as half the quantity of nutrients. A total of 88 plants was grown with 22 plants for each of the four treatments.

### Plant traits

#### Plant growth and leaf sampling

We measured the plant height and counted the number of nodes per plant on 34 DAS.

To characterize the defense chemistry, the plants in two groups were sampled for the subsequent glycoalkaloids quantification (Fig. 5): (1) “Without Tuta”, leaf samples were collected on 48 DAS from *T. absoluta*-free plants with constitutive defense. Twelve plants were sampled, with three plants from each of the four treatments (n = 3); (2) “With Tuta”: leaf samples were collected from the *T. absoluta*-infested plants on 48 DAS when infested *T. absoluta* started to pupate, i.e., induced defense. Seventy-six plants in all were sampled with 19 plants from each of the four treatments (n = 19). We sampled all the leaves by cutting the fourth fully-developed leaves from the apex, the ones next to the third leaves that had been used for insect infestation (see below “*Insect infestation*”). The leaf samples were dried in a oven at 60 °C for 72 h and kept for further glycoalkaloid analyses.

#### Glycoalkaloid analyses

Glycoalkaloids act as key defensive compounds against various herbivorous insects in most plants from the Solanaceae family, e.g., tomato^47^,^48^,^52^,^53^. Glycoalkaloids were extracted from 5 mg dried tomato leaf powder mixed with 2 mL of 5% acetic acid (CH_3_COOH) in water (v/v). The suspension was first mixed by vortexing and then extracted twice for 30 min using an ultrasonic assisted extractor at room temperature. After the extraction, the supernatant was filtered through a 0.45 μm PVDF PuradiscTM (Whatman, GE Healthcare). All samples were kept at −20 °C until analyzed. Glycoalkaloid standards (α-tomatine and tomatidine; Extrasynthese, Genay, France) were also diluted in a 5% CH_3_COOH solution.

All analyses were performed on an Ultimate 3000 Rapid Separation LC (RSLC) system (Thermo Scientific) equipped with a PDA detector and coupled to an ESI-Q-TOF mass spectrometer (microTOFQII, Bruker Daltonics). Separation was carried out on an Ascentis Express Fused-Core™ C18 column (100 × 2.1 mM i.d., 2.7 μm; Supelco) with its corresponding guard column (Ascentis express, 2.1 mM id x 50 mM, 2.7 μm, Supelco). An elution gradient was developed to separate glycoalkaloids. The flow rate was set at 400 μL/min and the solvent system was (a) water (H_2_O) and formic acid (FA, 0.1% v/v) and (b) acetonitrile (ACN) 0.1% FA (v/v). The elution program was: 2% b for 5 min, 50% b for 35 min, 100% b for 5 min and thermostated for 3 min, back to 2% b in 5 min and conditioning for 2.5 min. The column oven was thermostated at 35 °C and the auto sampler at 6 °C. The injection volume was set at 5 μL.

Before analysis, the mass spectrometer was calibrated in an external mode using a mix of known masses (ESI-L Low concentration Tuning Mix, Agilent Technologies). HRMS data were acquired in positive ionization and in MS scan modes. The source temperature was set at 195 °C, the capillary voltage at 3.8 kV, nebulizer gas (N_2_) at 2.8 bars and dry gas (N_2_) at 9 L/min. Mass spectra acquisition was set at 5000 spectra/sec on a mass range of 50–2000 m/z. LC-MS raw data were processed using Data Analysis 4.1 software (ESI Compass 1.5, Bruker Daltonique).

The two targeted glycoalkaloids α-tomatine and tomatidine were observed respectively at m/z 1034.5550 and m/z 416.3543. However, injection of α-tomatine produced two different peaks (α-tomatine 1 and 2, see Supplementary Table S1) on our LC-MS platform which exhibited different retention times but a similar pseudo-molecular ion and fragmentation pattern. Furthermore, dehydrotomatine was also observed in tomato leaf samples at m/z 1032.5377 and characterized by a typical fragment ion corresponding to [Tomatidenol+Gal+H]+ at m/z 576.3876 as described by Cataldi *et al*.^53^.

An ion extraction method using a mass range of 0.01 Da was used to quantify these four glycoalkaloids. To obtain the corresponding quantity in μg of compounds per mg of leaf dry mass, the measured ion abundance was reported on a calibration curve for α-tomatine and tomatidine, obtained from the same analysis and reprocessing conditions. Since a standard for dehydrotomatine was not commercially available, we could not calculate the quantities of this compound in the leaves. A relative quantification of this compound was then given in ion abundance per mg of leaf dry mass.

### Insect infestation

On 30 DAS, the terminal leaflet of the third fully-developed leaf from the apex in each plant was chosen to be infested with one newly-oviposited *T. absoluta* egg, i.e., ≤24 h (Fig. 5). Nineteen plants were infested for each of the four treatments (n = 19). The eggs were monitored for the following four days since they took an average of four days to hatch under laboratory conditions (16 h light, 2 5± 1 °C, 60 ± 5% RH)^10^. If the eggs failed to hatch due to unintentional damage during the transfer, newly-hatched larvae were placed on the leaflets. To avoid larvae escaping, a plastic arena made from a 9-cm diameter petri-dish was used to trap each infested leaf. A 6-cm diameter hole on one side of the arena was covered by a 0.2 mM nylon mesh allowing ventilation. Such an arena design has been successfully used in our previous studies^33^,^51^.

### Insect traits

The infested leaves were detached on 48 DAS, the same date as the leaf sampling for the glycoalkaloids measurement. The detached leaves with *T. absoluta* pre-pupae or pupae were maintained by inserting the stem into the sponge substrate saturated with water to maintain moisture for the insects. When all the individuals completely pupated, the leaves were removed and a small cotton ball saturated with water was placed in the arena to retain moisture. To estimate *T. absoluta* survival rate, the number of the individuals reaching pupal or adult stage was recorded. Pupal weight of each individual was measured when it had pupated completely. The development time from egg to pupa or to adult was recorded for each individual.

### Data analyses

We firstly used MANOVAs to test the effects of water (limited water *vs.* optimal water input), salinity (with *vs.* without addition of salt), and/or insect (presence *vs.* absence of *T. absoluta* infestation) on the complex of variables, i.e., two and more dependent variables. The results showed significant effects of water and/or salinity on all the complex of variables, except for glycoalkaloids data. Hence separate factorial ANOVAs performed on each variable appeared to be justified. We subsequently performed factorial ANOVAs to test the effects of “water”, “salt” and their interactions on plant height and number of nodes per plant independently. Likewise, factorial ANOVAs were conducted to examine the effects of “water”, “salt”, “insect” and their interactions, if applicable, on the four glycoalkaloids: tomatidine, α-tomatine 1, α-tomatine 2 and dehydrotomatine. Once a significant main effect of any factor was found in any trait, the differences among the four water and salinity treatments were tested using Tukey’s post hoc test for multiple comparisons.

The Chi-square test was used to examine the effects of “water” and “salt” on *T. absoluta* survival rates from egg to pupa or to adult. The factor “stage” was also tested to determine if *T. absoluta* survival responded differently to the treatments according to different stages. The survival data was further analyzed with the permuted Fisher exact test. Factorial ANOVAs were performed to test the effects of “water”, “salt” and their interactions on a) *T. absoluta* pupal weight, b) development time from egg to pupa and c) development time from egg to adult. Multiple comparisons within each trait were performed using Tukey’s post hoc test when the main effect was significant. All these data were computed using R software^54^.

## Additional Information

**How to cite this article**: Han, P. *et al*. Increased water salinity applied to tomato plants accelerates the development of the leaf miner *Tuta absoluta* through bottom-up effects. *Sci. Rep.* **6**, 32403; doi: 10.1038/srep32403 (2016).

## Supplementary Material

Supplementary Information

## Figures and Tables

**Table 1:** Factorial ANOVAs to test the effects of “water”, “salt”, “insect (*T. absoluta*)”, and their interactions (if applicable) on (**A**) plant traits: plant height and number of nodes per plant on 34 DAS (DAS - days after sowing), concentrations of four glycoalkaloids: tomatidine, α-tomatine 1 and α- tomatine 2 and dehydrotomatine; (**B**) insect traits: *T. absoluta* pupal weight, development time from egg to pupa and development time from egg to adult.

(A) Plant traits	Plant height		No. of nodes/plant		Tomatidine		α-tomatine 1		α-tomatine 2		Dehydrotomatine	
	F_1,84_	*P*	F_1,84_	*P*	F_1,80_	*P*	F_1,80_	*P*	F_1,80_	*P*	F_1,80_	*P*
water	**81.33**	**<0.001**	**49.68**	**<0.001**	**4.940**	**0.029**	1.476	0.228	2.168	0.145	2.326	0.131
salt	**64.41**	**<0.001**	**15.68**	**<0.001**	**8.264**	**0.005**	2.565	0.113	3.327	0.072	2.480	0.119
insect	**−**	**−**	**−**	**−**	1.278	0.262	0.314	0.577	0.436	0.511	0.349	0.557
water x salt	0.310	0.577	0.010	0.916	2.997	0.087	0.576	0.450	1.260	0.265	1.300	0.258
water x insect	**−**	**−**	**−**	**−**	0.707	0.403	0.108	0.744	0.199	0.657	0.249	0.619
salt x insect	**−**	**−**	**−**	**−**	1.184	0.280	0.336	0.564	0.593	0.444	0.420	0.519
water x salt x insect	**−**	**−**	**−**	**−**	0.489	0.486	0.154	0.696	0.333	0.566	0.285	0.595
**(B) Insect traits**	Pupal weight		Development time from egg to pupa					Development time from egg to adult				
Source of variation	F_1,56_	*P*	F_1,56_				*P*	F_1,42_				*P*
water	**7.169**	**0.010**	2.258				0.139	3.179				0.082
salt	0.253	0.617	**8.789**				**0.004**	**6.607**				**0.014**
water x salt	1.206	0.277	1.737				0.193	1.717				0.197

The statistical results of effects on *T. absoluta* survial are presented in the text.
