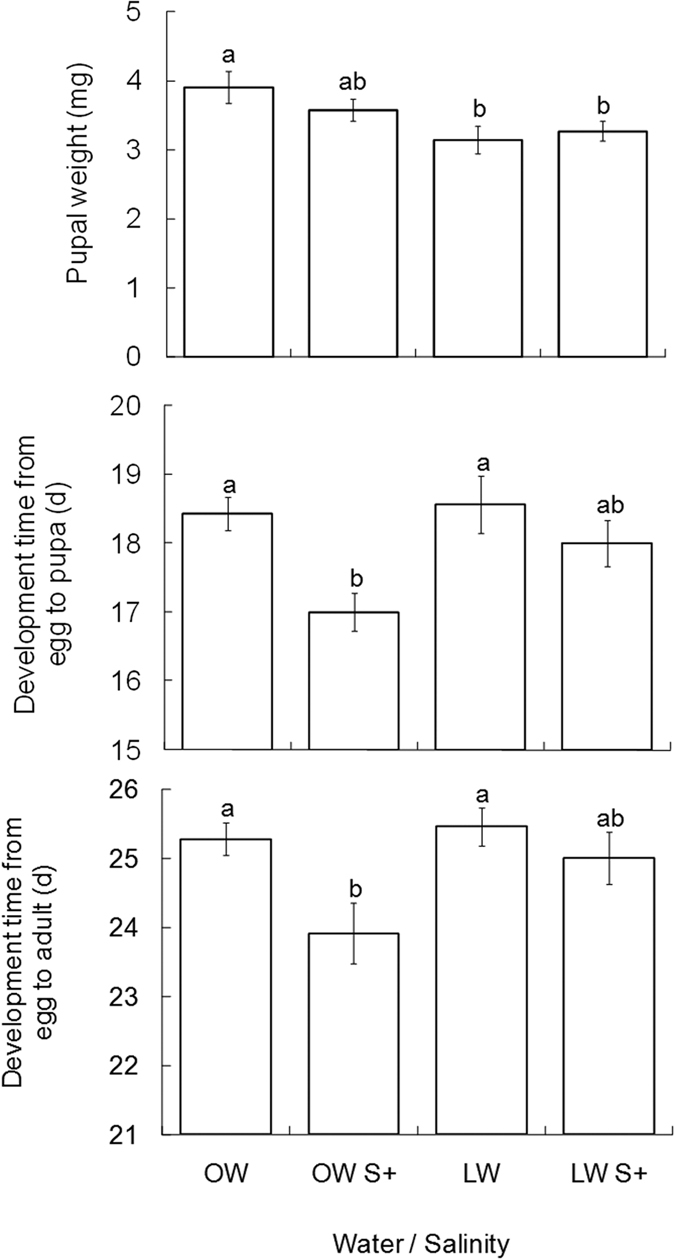# Corrigendum: Increased water salinity applied to tomato plants accelerates the development of the leaf miner *Tuta absoluta* through bottom-up effects

**DOI:** 10.1038/srep39037

**Published:** 2016-12-13

**Authors:** Peng Han, Zhi-jian Wang, Anne-Violette Lavoir, Thomas Michel, Aurélie Seassau, Wen-yan Zheng, Chang-ying Niu, Nicolas Desneux

Scientific Reports 6: Article number: 32403; 10.1038/srep32403 published online: 09 13 2016; updated: 12 13 2016

This Article contains typographical errors. In the Results section under subheading ‘*T. absoluta* development’,

“The average pupal weight of the individual feeding on plants treated with OW, OW S+, LW and LW S+ was 3.91 g, 3.58 g, 3.15 g and 3.27 g, respectively”.

should read:

“The average pupal weight of the individual feeding on plants treated with OW, OW S+, LW and LW S+ was 3.91 mg, 3.58 mg, 3.15 mg and 3.27 mg, respectively”.

In Figure 4, the y-axis ‘Pupal weight (mg)’ is incorrectly given as ‘Pupal weight (g)’. The correct Figure 4 appears below as Figure 1.

## Figures and Tables

**Figure 1:**