# Supplementary material for: Increased water salinity applied to tomato plants accelerates the development of the leaf miner Tuta absoluta through bottom-up effects
Source: Sci Rep. 2016 Sep 13;6:32403. doi: 10.1038/srep32403 (PMC5020321; doi:10.1038/srep32403)
Supplement: Supplementary Information [file srep32403-s1.doc]

Submitted to ***Scientific Reports –* R2**

**Increased water salinity applied to tomato plants accelerates the development of the leaf miner *Tuta absoluta* though bottom-up effects**

Peng Han^*^, Zhi-jian Wang, Anne-Violette Lavoir, Thomas Michel, Aurélie Seassau, Wen-yan Zheng, Chang-ying Niu, Nicolas Desneux^*^

*** Corresponding author:**

**PH:** [penghan1394@gmail.com](mailto:penghan1394@gmail.com)

**ND:** [nicolas.desneux@sophia.inra.fr](mailto:nicolas.desneux@sophia.inra.fr)

**Table S1: Chemical details for glycoalkaloid compounds.**

| **Rt (*min*)** | **Compound name** | **Chemical**  **formulae** | **Accurate mass Measured (*m/z*)** | **In-source Fragmentation**  **(*m/z*)** |
| --- | --- | --- | --- | --- |
| 21.3 | α-tomatine 1 | C_50_H_83_NO_21_ | 1034.5550  [M+H]^+^ | 578.4062  526.2973  416.3509  295.1042 |
| 22.1 | α-tomatine 2 | C_50_H_83_NO_21_ | 1034.5552  [M+H]^+^ | 78.4066  526.2976  416.3520  295.1043 |
| 27.7 | tomatidine | C_27_H_45_NO_2_ | 416.3543 [M+H]^+^ | / |
| 20.7 | dehydrotomatine | C_50_H_81_NO_21_ | 1032.5377  [M+H]^+^ | 576.3876  525.2860 |
